# Chylous Ascites, Unusual Association with Ductal Pancreatic Adenocarcinoma with Plasmacytoid Morphology: A Case Report and Literature Review

**DOI:** 10.1055/s-0041-1728651

**Published:** 2021-07-19

**Authors:** Catalin Bogdan Satala, Tivadar Jr. Bara, Ioan Jung, Vlad Tudorache, Simona Gurzu

**Affiliations:** 1 Department of Pathology, George Emil Palade University of Medicine, Pharmacy, Sciences and Technology, Targu-Mures, Romania; 2 Department of Surgery, George Emil Palade University of Medicine, Pharmacy, Sciences and Technology, Targu-Mures, Romania; 3 Research Center for Oncopathology and Translational Medicine, George Emil Palade University of Medicine, Pharmacy, Sciences and Technology, Targu-Mures, Romania; 4 Department of Gynecology, Clinical County Hospital, Targu-Mures, Romania

**Keywords:** chylous ascites, pancreatic neoplasms, lymph node, plasmacytoid

## Abstract

Chylous ascites represents a relatively uncommon condition. In this paper, we present a case of chyloperitoneum associated with pancreatic ductal adenocarcinoma (PDAC) and a review of literature regarding chylous ascites. A 76-year-old male patient was admitted in emergency department with acute abdomen. A pancreatic cancer was suspected. Subtotal spleno-pancreatectomy, for a nodular mass infiltrating the mild and distal portion of the pancreas, was necessary. During surgical intervention in the peritoneal cavity, a moderate quantity of whitish and thick consistency fluid with milk-like appearance was observed to be accumulated. After examination of the fluid, chyloperitoneum was diagnosed. The histologic examination showed a PDAC, with multiple emboli in lymph vessels, with tumor cells with plasmacytoid morphology, diagnosed as lymphangiosis carcinomatosa. The patient died at 3 weeks after surgical intervention. In patients with pancreatic cancer and chylous ascites, suspicion of tumor-related blockage of the lymphatic flow should be suspected. Prognosis of PDAC should be evaluated not only based on the number of lymph node metastases, but also considering the number of lymph vessels with tumor emboli and the architecture of tumor cells. This is the first reported case of a PDAC with plasmacytoid morphology of lymphangiosis carcinomatosa.

Chylous ascites, also known as chyloperitoneum or lymphorrhagia, is a rare lesion of adults, with a reported incidence of 1:20,500, which is defined by the presence of a milky and lipid-rich fluid in the peritoneal cavity. 1 2 3 4 5 6

Lymphatic fluid is characterized by thick consistency, cell count >500/mL, with lymphocytic predominance, total protein level between 2.5 and 7.0 g/dL, with serum to ascites albumin gradient (SAAG) less than 1.1 g/dL, lactate dehydrogenase (LDH) between 110 and 200 IU/L, glucose level >100 mg/dL, and triglyceride level in ascetic fluid >110 mg/dL. 1 2

In this paper, we present an unusual case of a ductal carcinoma of the pancreatic tail (PDAC) associated with chylous ascites, as result of tumor emboli-related blockage of the lymphatic flow. A review of literature was also done.

## Case Report

A 76-year-old male was admitted to the general surgery department with 1-month history of abdominal pain, weight loss (5 kg in the last month), accusing nausea, and multiple episodes of emesis. He was a social drinker and he quitted smoking 2 years ago. He also related to be treated for arterial hypertension.

At the present admission, the tenderness painful abdomen was diagnosed as acute abdomen. The ultrasound examination showed pancreatic enlargement and suspicion of a pancreatic cancer was done. The computed tomography (CT scan) with contrasting substance revealed that a pancreatic nodular mass, larger than 50 mm which infiltrate the pancreatic body and tail, was extended in the splenic hilum and associated multiple supra- and subdiaphragmatic adenopathy.

Bilateral deep vein thrombosis of the femoral and popliteal veins were associated and interpreted as Trousseau's sign. Emergent laparotomy was decided.

The signed informed consent for surgical intervention and publication of data was obtained before surgery.

The exploratory laparotomy revealed an infiltrative tumor mass of the mild portion of the pancreas, penetrating the celiac trunk, inferior mesenteric vein, and spleno-portal confluent, with peripancreatic adenopathy. A moderate quantity of whitish and milky-like fluid was identified in the peritoneal cavity and was collected for biochemistry examination.

Subtotal spleno-pancreatectomy was performed, with the suture of celiac trunk, inferior mesenteric, and portal vein. The surgical specimens were sent for histopathological examination.

Laboratory examination of the peritoneal fluid revealed 295 mg/dL triglycerides, more than 500 cells/mL, with the majority of them being lymphocytes, a protein count of 5.4 g/dL, with SAAG of 0.94 and LDH of 187 IU/dL. The glucose level of the abdominal fluid was not quantified. With five out of six diagnostic criteria achieved, the diagnosis of chylous fluid was established.

Macroscopic examination of the surgical specimens revealed a 50 × 40 × 40-mm white and infiltrative mass involving the middle and distal portion of the pancreas, with direct infiltration of the spleen hilum, free pancreatic lateral and posterior margin and crossing of the pancreatic anterior margin. Multiple tumor deposits were also identified in the peripancreatic tissue.

**Fig. 1:**
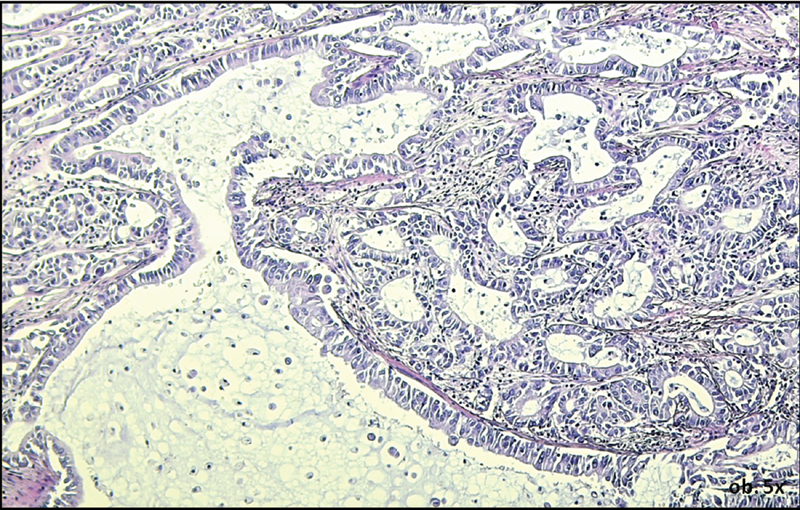
The histologic examination of the pancreatic ductal adenocarcinoma reveals in hematoxylin eosin, tubular structures located in a poorly defined stroma.

**Fig. 2:**
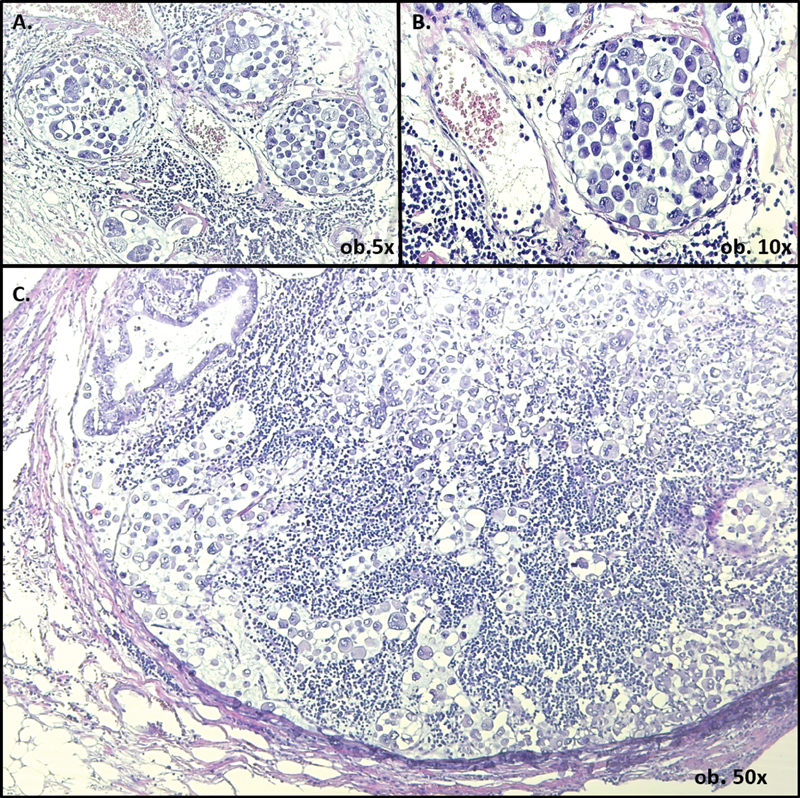
Lymphangiosis carcinomatosa is defined by the presence of multiple tumor emboli within the dilated lymph vessels ( **A, B** ). Examination of the metastatic lymph nodes reveals poorly defined tubular structures and highly pleomorphic dyscohesive tumor cells with plasmacytoid morphology ( **C** ).

Microscopically, the well-defined tubular structures, some of them being hugely dilated, alternated with poorly differentiated areas ( Fig. 1 ). The tumor cells proved positive for carcinoembryonic antigen and maspin, and negative for chromogranin, synaptophysin, and neuroendocrine cluster of differentiation (CD56) and S100. Invasion of over 10 of the peripancreatic and spleen hilum lymph nodes conglomerates associates perineural invasion and presence of tumor emboli in over 20 of the significantly enlarged lymph vessels. In the metastatic tissue and emboli, the tumor cells were dyscohesive and showed high pleomorphism ( Fig. 2 ). The metastatic discohesive cells showed plasmacytoid morphology, with eccentrically located nuclei, indistinct nucleoli, and eosinophilic cytoplasm. 7

The microscopic features, correlated with the immunoprofile of the tumor cells and clnicopathological characteristics, allowed the diagnosis of a pathologic tumor-node stage III (pT4N2) poorly differentiated PDAC with lymhangiosis carcinomatosa (carcinomatosis of lymph vessels) and chyloperitoneum.

The postoperative evolution was unfavorable, the chylous ascites was persistent, despite persistent drainage, and patient died at 3 weeks after surgery.

## Discussion

In children, congenital abnormalities and traumatic disruption of the lymph vessels are the commonest causes of chylous ascites. In adults, this is an extremely rare lesion, which can be related on tumor and nontumor lesions ( Table 1 ).

**Table 1:** Etiology and mechanisms of occurrence of chylous ascites

	Etiology	Mechanism
**Acquired**	Mechanic, traumatic agents	Obstruction or disruption of main lymph vessels
	Surgical technique imperfections	
	Infectious agents with lymph vessels tropism (tuberculosis, filariasis)	
	Autoimmune diseases (systemic lupus erythematous)	
	Cirrhosis	Lymph overflow
	Cardiac diseases	
	Tumors (lymphomas, pancreatic ductal adenocarcinoma, gastrointestinal neuroendocrine tumors/adenocarcinomas, sarcomas)	Fibrosis of lymph vessels and/or lymph nodes
	Radiotherapy	
**Congenital**	Lymphangiectasia	Congenital malformations
	Lymphangioma	
	Congenital syndromes (such as Klippel-Trenaunay syndrome)	

In developed, western countries, abdominal tumors, cirrhosis, and iatrogenic lymph vessels disruption during surgery accounts for more than half of chylous ascites, while in developing countries, the most frequent etiologies responsible for this condition are infectious diseases like tuberculosis and filariasis. 1 2

The main mechanisms of acquired chylous ascites are the following ones: traumatic disruption of thoracic duct, obstruction of lymphatic flow, caused by infectious agents or tumor cells, fibrosis of the lymph vessels wall, in patients with autoimmune diseases or after radiotherapy, or increased lymph fluid production, such in cirrhosis or cardiovascular diseases. 3 6 8 9 10

Although rare, malignancies such as lymphomas (30% of all reported cases), Kaposi sarcoma, leukemias, neuroendocrine tumors or adenocarcinomas of the gastrointestinal tract, and carcinomas of lung and endometrium were described to be the possible causes of chylous ascites. 1

As regarding the pancreas, chylous ascites was reported as a rare complication of iatrogenic lesions of the lymphatic channels during cephalic duodenopancreatectomy with extensive lymphadenectomy. 2 Postoperative lymphorrhagia is usually reversible within few days, spontaneously or after administration of somatostatin analogues or octreotide. 11 Total parenteral nutrition associated with diuretics, medium chain triglycerides, and removal of the drainage tube was also suggested. 2 12

Even though it is not the most common cause of chylous ascites, PDAC can cause it via lymphangiosis carcinomatosa, respectively obstruction and disruption of lymphatic channels flow. Representing one of the most lethal tumors worldwide, PDAC is the 12th most common encountered cancer worldwide, with an incidence of 4.2 per 100,000 and over 330,400 deaths per year. 13 14 Fewer than 20% of PDACs prove to be surgically resectable at the time of diagnosis. 13

As the lymph node metastasis is the most significant prognostic factor of PDAC, in a recent study, the possible prognostic role of the degree of lymphatic vessel invasion was emphasized. 14 It was proved that except lymph node metastasis, presence of tumor emboli in at least five lymphatic vessels is a negative prognostic factor of locally advanced surgically resectable PDAC of the pancreatic head. Moreover, presence of emboli in at least nine lymphatic vessels, like our case, were comparable to or surpassed the significance of lymph node metastasis. 14 In patients without lymph node metastases but tumor cells in at least 10 lymphatic vessels, the survival rate was similar with those having positive lymph nodes. 14

A grading system of lymphatic invasion (ly) was proposed by the Japan Pancreatic Society, which suggested to be used in daily diagnosis and classify cases based on the following four-tier code: ly0 (no evidence of invasion), ly1 (slight invasion), ly2 (moderate invasion), and ly3 (marked invasion). 14 15

Besides the lymphangiosis carcinomatosa, another aggressive indicator was the histological aspect of the tumor cells. The primary tumor showed tubular structures, but plasmacytoid morphology was seen in the tumor emboli and lymph node metastases. Although rare, gastroenteropancreatic plasmacytoid carcinomas are considered high grade tumors, which aggressivity seems to be induced by the epithelial mesenchymal transition (EMT) phenomenon revealed by loss of E-cadherin and nuclear translocation of β-catenin due to alteration of the Wnt/β-catenin pathway. 7 16 17 It was recommended to call these tumors as “mesenchymal-type poorly cohesive carcinomas” 7 . Only 10 cases of gastrointestinal carcinomas with plasmacytoid appearance were reported till 2020: seven of the stomach, two of the ampulla of Vater, and one in the rectum. 7 16

In pancreas, plasmacytoid morphology was described in cytologic specimens, in “lipid-rich variant of pancreatic endocrine tumors” 18 , fine needle aspirations of serous cystadenomas, 19 and clear cell variant of solid pseudopapillary neoplasms (SPN). 20 21 To our knowledge, no cases of PDAC with plasmacytoid features and chylous ascites were described yet. As the present case involved the distal part of the pancreas like the SPN, and the later type is mainly developed in the pancreatic tail as result of the Wnt/β-catenin pathway disruption, 17 21 it can be supposed that the PDAC with plasmacytoid component can be an aggressive variant of pancreatic SPN.

Independently by localization, the plasmacytoid carcinomas are known to be predisposed to intraperitoneal spread and carcinomatous ascites 7 22 like our case. In patients with PDAC, the amount of chylous fluid is usually small preoperatively and CT scan might fail to identify it. In suspected cases, diagnosis is mainly based on lymphoscintigraphy with ^99m^ Technetium-labeled human albumin. 8 If it is detected preoperatively, the case is usually inoperable and peritoneo-venous shunt is the therapy of choice. 23 To decrease the secretion of chylous fluid, the administration of furosemide and somatostatin analogs or octreotide—with/without etilefrine—is indicated. 11 23 Low-fat diet, total parenteral nutrition, or other conservative methods are also recommended. 11 23 In these patients, however, after shunting, the quality of life or survival period are not significantly improved.

The present case highlights the need for a more attentive preoperative evaluation of patients with PDAC and at the same time, the negative role of associated chylous ascites, in these patients.

No data were published yet regarding the relation between the number of involved lymphatic vessels and risk of chylous ascites even about the minimum number of lymphatic vessels with tumor emboli that can be used as a negative prognostic factor, in patients with PDAC of the pancreatic tail.
